# Argumentation und Narration in verschwörungstheoretischen Youtube-Videos

**DOI:** 10.1007/s41244-021-00203-5

**Published:** 2021-04-14

**Authors:** Thomas Niehr

**Affiliations:** Institut für Sprach- und Kommunikationswissenschaft, RWTH Aachen, Aachen, Deutschland

**Keywords:** Argumentation, Geltungsanspruch, Narration, Konklusive Sprechhandlung, Politik, Politolinguistik, Rhetorik, Verschwörungstheorie, Argumentation, Claim of Validity, Narrative, Conclusive Speech Acts, Politics, Political Linguistics, Rhetoric, Conspiracy Theory

## Abstract

In diesem Beitrag wird das Verhältnis von Argumentation und Narration in sogenannten Verschwörungstheorien näher beleuchtet. Im Anschluss an die in der klassischen Rhetorik beschriebene Funktion der *narratio* in einer Gerichtsrede wird der Frage nachgegangen, in welcher Weise narrative Elemente bei verschwörungstheoretischen Argumentationen eingesetzt werden und welche Funktionen sie dabei übernehmen. Am Beispiel ausgewählter Youtube-Videos des Verschwörungstheoretikers Heiko Schrang wird illustriert, wie narrative Elemente eine Argumentation stützen und Rezipient*innen Anschluss an die eigenen Wissensbestände sichern können.

## Abstract (en)

In this contribution, the relationship between argumentation and narration is examined in more detail. Following on from the function of narration in a court speech as described in classical rhetoric, the question is examined as to how narrative elements are used in conspiracy theory arguments and what functions they assume in the process. Using the example of selected YouTube videos by the conspiracist Heiko Schrang, we will illustrate how narrative elements can support an argumentation and ensure that the recipient is connected to his or her own body of knowledge.

## Das Verhältnis von Argumentation und Narration

Das Verhältnis von Argumentation und Narration wird seit relativ kurzer Zeit in der Linguistik, der Literaturwissenschaft und Rhetorik verstärkt in den Blick genommen.^1^ Dies mutet umso erstaunlicher an, als ja in der antiken Rhetorik die *narratio* als fester Bestandteil der (Gerichts‑)Rede gilt (vgl. Bleumer/Hannken-Illjes/Till 2019b, S. 7–9; Girnth/Burggraf 2019a, S. 565–571, 2019b, S. 109 f.; Ottmers 1996, S. 56 f.). Ihr kommt eine wichtige »Scharnierfunktion« zu (Till 2019, S. 124), weil sie die »Basis des sich anschließenden Beweisverfahrens« ist (Ottmers 1996, S. 57) und ihr damit die Aufgabe zukommt, »das Publikum [...] von der Wahrhaftigkeit des Erzählten zu überzeugen« (Ottmers 1996, S. 57):

Einen besonderen Stellenwert für die Überzeugungskraft einer Erzählung nimmt dabei die Glaubwürdigkeit des geschilderten Geschehens ein. Werden die Handlung sowie die Charaktere von Seiten der Adressaten als stimmig und plausibel empfunden, steigt die Wahrscheinlichkeit, dass diese die narrativen Ereignisse während der Rezeption mithilfe ihrer Vorstellungskraft intensiv miterleben. (Girnth/Burggraf 2019a, S. 569)

Offensichtlich erscheint vor diesem Hintergrund, dass der *narratio* eine Schlüsselfunktion für die Argumentation einer Rede zukommt. Dass *narrative Elemente* auch bei politisch motivierten Argumentationen eine wichtige Rolle spielen dürften, ergibt sich aus mehreren Gründen.

Erstens geht es in den meisten politischen Kontexten darum, für eine bestimmte (perspektivisch-ideologische) Sicht der Dinge – oder konstruktivistischer gesprochen: der Problemverhalte^2^ – zu werben. Hermann Lübbe (1975, S. 107) definiert in diesem Sinne Politik als »die Kunst, im Medium der Öffentlichkeit Zustimmungsbereitschaften zu erzeugen«. Eine derartige Kunst erschöpft sich nun allerdings keineswegs darin, möglichst rationale Argumente in möglichst großer Zahl aneinanderzureihen und dadurch das angestrebte argumentative Ziel – in diesem Fall Zustimmung zu bestimmten politischen Maßnahmen – zu erreichen. Dies ist der fundamentale Unterschied zwischen Wissenschaft und Politik. Vielmehr muss die politische Überzeugungsarbeit auch »die Herzen der Menschen« erreichen, mithin emotionale Zustimmung hervorrufen. Hier ist die wichtige Rolle der *narratio* bzw. narrativer Elemente zu sehen. Denn die narratio ist »nicht derjenige Teil der Rede, der dem Beweis bzw. der Widerlegung dient« (Till 2019, S. 124). Diesen dient sie jedoch als Vorbereitung, »indem sie durch die parteiliche, also bewusst einseitige Darstellung des Streitfalles eine Ausgangsbasis für die eigentliche Beweisführung schafft« (Till 2019, S. 124). Quintilian spricht im Zusammenhang der Gerichtsrede von der Darlegung des Falles und führt aus:

Sie [die *narratio*, Th. N.] kann nicht nützlich sein, wenn nicht vorher feststeht, was man für die Beweisführung versprechen kann. Schließlich muss man darauf schauen, wie man den Richter für sich gewinnen kann. Denn erst, wenn wir den Fall in allen seinen Teilen genau überblicken, können wir wissen, wie es sich empfiehlt, den Richter zu stimmen, wenn er ihn kennenlernt, streng oder milde, gereizt oder gelassen, Persönlichem gegenüber abweisend oder empfänglich. (Quintilian 2015, III, 9, S. 7)

Der hier angesprochene Aspekt, eine Person für sich zu gewinnen bzw. positiv zu stimmen, wird von Quintilian an anderer Stelle noch einmal explizit hervorgehoben:

Selbst mein Satz, die Erzählung eines Sachverhaltes, den der Richter schon kennt, sei überflüssig, ist nicht ohne Einschränkung aufzufassen: ich möchte es so verstanden wissen: sie ist überflüssig, falls der Richter nicht nur weiß, was geschehen ist, sondern auch die Auffassung hat, es sei so geschehen, wie es uns zustattenkommt. Denn die Erzählung ist ja nicht dazu erfunden, daß der Richter eine Sache nur kennenlernt, sondern weit mehr dazu, daß er ihr zustimmt. (Quintilian 2015, IV, 2, S. 20 f.)

Es geht mithin bei der *narratio* darum, das Publikum für sich zu gewinnen, das Publikum darauf einzustimmen, den zur Verhandlung stehenden Fall auf eine spezielle Art und Weise zu betrachten und die vom Redner präsentierte Version als stimmig zu akzeptieren. Das Publikum soll also dazu gebracht werden, eine bestimmte – perspektivische – Lesart zu übernehmen und als gültig zu ratifizieren. In der *narratio* kommt also – um die bühlerschen Kategorien zu bemühen – insbesondere die Ausdrucks- und Appellfunktion sprachlicher Zeichen zur Geltung.

Eine derartige Einstimmung wird heutzutage in politik- und kommunikationswissenschaftlichen Kontexten gerne als *Framing* bezeichnet.^3^ Es handelt sich dabei um das Setzen eines Deutungsrahmens. Dieser bestimmt – sofern das Framing erfolgreich ist – die Perspektive, unter der der zur Verhandlung stehende Problemverhalt von den Rezipient*innen wahrgenommen wird. Gleichzeitig damit wird die grundlegende Einstellung des Publikums gegenüber eben diesem Problemverhalt gesteuert bzw. zu steuern versucht. Ein so verstandenes Framing ist ein wichtiger Bestandteil politischer Kommunikation. Diese muss nicht nur rational überzeugen, sie enthält auch Elemente narrativer Persuasion. Politik lässt sich in diesem Sinne auch charakterisieren als »die Kunst, die Einstellungen seines Publikums mithilfe von Erzählungen zu beeinflussen« (Girnth/Burggraf 2019b, S. 107).

Ein zweiter Grund, Narration und Argumentation bei politischen Argumentationen zusammenzudenken, ist darin zu sehen, dass antike Rhetorik wie auch zeitgenössische Autoren die Rede bzw. Argumentation vor Gericht als Musterbeispiel ansehen. Die *narratio* ist die »Schilderung des Tathergangs aus der Sicht des Anklägers oder des Verteidigers« (Ottmers 1996, S. 56). Es ist kein Zufall, dass auch Toulmin in seinem Standardwerk *Der Gebrauch von Argumenten* Argumentation immer wieder in juristischen Analogien denkt. Zentral für seine Theorie ist eine Feststellung, die er gleich am Beginn seiner Ausführungen trifft: »Der in einer Behauptung enthaltene Anspruch ähnelt einem Rechtsanspruch.« (Toulmin 1996, S. 17)^4^ Diesen gilt es durchzusetzen oder zu verteidigen. Das bevorzugte Mittel dazu ist die Argumentation, innerhalb derer wiederum narrative Bestandteile als Hilfsmittel verwendet werden können, um für die eigene Position zu werben.^5^ Da auch politische Kommunikation zu weiten Teilen werbende Kommunikation ist^6^, sind die strukturellen Ähnlichkeiten zwischen politischer Kommunikation und der vor Gericht unübersehbar: Hier wie dort geht es darum, »Ansprüche« durchzusetzen, für die Zustimmung des Publikums zu den je eigenen Positionen zu werben. Dies wird durch die entsprechende Einstimmung mittels perspektivischer Darstellung unterstützt. Auf diese Weise wird also versucht, einer möglicherweise notwendig werdenden Argumentation gute Ausgangsbedingungen zu verschaffen.

## Verschwörungstheorien

Den Ausdruck *Verschwörungstheorie* in einer wissenschaftlichen Abhandlung zu verwenden, kann sich als problematisch erweisen. Dies hängt mit seiner pejorativen Bedeutung zusammen, die eher an die (politisch motivierte) Abwertung eines missliebigen Standpunkts bzw. einer kritikwürdigen Haltung denken lässt als an eine durch Rationalität gekennzeichnete Auseinandersetzung.^7^ Dieses Schicksal teilt *Verschwörungstheorie *mit anderen Termini wie *Schlagwort* oder *Leerformel*. Auch sie werden in wissenschaftlichen Kontexten verwendet und kommen gleichzeitig mit pejorativer Bedeutung in der Alltagssprache vor. Deshalb bedarf es jeweils einer exakten Begriffsdefinition, um eine Verschmelzung von (pejorativer) Alltagsbedeutung und möglichst wertungsfreier wissenschaftlicher Bedeutung zu vermeiden.^8^ Aus diesem Grunde wird zunächst erläutert, in welcher Bedeutung *Verschwörungstheorie* im Folgenden verwendet wird.^9^

Verschwörungstheorien behaupten, dass eine im Geheimen operierende Gruppe, nämlich die Verschwörer, aus niederen Beweggründen versucht, eine Institution, ein Land oder gar die ganze Welt zu kontrollieren oder zu zerstören. (Butter 2018, S. 21)

Mit dem Ausdruck *behaupten* in diesem Zitat werden implizit zwei Merkmale von Verschwörungstheorien angesprochen. Zunächst wird die Nähe zur Argumentation deutlich: Mit jeder Behauptung wird ein Geltungsanspruch (z. B. auf Wahrheit oder Wahrhaftigkeit) erhoben. Werden derartige Geltungsansprüche in Zweifel gezogen, so besteht die Notwendigkeit einer argumentativen Aushandlung bzw. Klärung.^10^ Ein wesentliches Merkmal von Verschwörungstheorien besteht nun gerade darin, dass sie gängige Deutungsmuster (und damit erhobene Geltungsansprüche) infrage stellen, indem sie andere, häufig konträre Geltungsansprüche erheben. Somit ergibt sich die Notwendigkeit einer argumentativen Legitimierung der behaupteten Verschwörung, zumindest wenn der Anschein von Rationalität gewahrt bleiben soll.

Weiterhin ergibt sich aus dem bisher Gesagten, dass Verschwörungstheorien versprachlicht werden müssen, um einen intersubjektiven Geltungsanspruch zu erheben.^11^ Sie treten üblicherweise in Form einer Erzählung, einer Narration^12^ auf, deren Funktion darin besteht, die behauptete Verschwörung zu plausibilisieren. Nicht nur aus diesem Grunde wird im Folgenden der gängige Ausdruck *Verschwörungs****theorie*** mehrfach durch *Verschwörungs****erzählung*** ersetzt. Damit soll einerseits die Bedeutung der *narratio* bzw. der narrativen Elemente bewusst gehalten werden, andererseits wird durch diesen Ausdruck der Tatsache Rechnung getragen, dass es sich bei Verschwörungserzählungen eben nicht um das handelt, was in der Wissenschaft üblicherweise als *Theorie* bezeichnet wird, nämlich ein »System wissenschaftlich begründeter Aussagen zur Erklärung bestimmter Tatsachen od. Erscheinungen u. der ihnen zugrunde liegenden Gesetzlichkeiten« (Duden 2019, S. 1786).^13^

Ein Charakteristikum von Verschwörungserzählungen kann darin gesehen werden, dass sie nicht nur eine Erzählung präsentieren, sondern sich häufig auf gängige Wirklichkeitsbeschreibungen beziehen, denen sie eine Alternative gegenüberstellen.^14^ Nach Seidler zeichnen sich Verschwörungstheorien geradezu dadurch aus, jeweils zwei Plots zu präsentieren, einen sichtbaren und einen unsichtbaren:

Verschwörungstheorien beziehen sich beim Erzählen des sichtbaren Plots ausnahmslos auf ein bereits bestehendes Narrativ, das sie unter der Annahme einer »totalen Verschwörung« neu zu erzählen suchen. [...] Der sichtbare Plot ist unverzichtbarer Bestandteil jeder verschwörungstheoretischen Erzählung. Der geheime, unsichtbare Plot der Verschwörung wird nur dadurch erzählbar, dass [...] »Defekte« im sichtbaren Plot aufgedeckt werden. (Seidler 2016, S. 35 f.)

Dass Aufdecken derartiger »Defekte« in einem Plot ist aus argumentationsanalytischer Sicht nichts anderes als das Bestreiten von Geltungsansprüchen. Dies aber macht eine Argumentation geradezu erwartbar. Deren *quaestio* besteht darin, den mit einem verbreiteten (»sichtbaren«) Plot jeweils erhobenen Geltungsanspruch (»So hat es sich zugetragen.«) zu prüfen (»Hat es sich tatsächlich so zugetragen?«) bzw. zu widerlegen (»So hat es sich keineswegs zugetragen! Tatsächlich ist Folgendes geschehen.«) Der Zusammenhang zwischen (verschwörungstheoretischer) Argumentation und Narration ist also naheliegend^15^ und soll im Folgenden an Beispielen exemplifiziert werden. Ein beim Verfassen dieses Aufsatzes aktuelles Beispiel liefern Verschwörungserzählungen, die sich auf die Corona-Krise beziehen. Letztere liefert Stoff für zahlreiche Verschwörungserzählungen.^16^ Diese gehen etwa davon aus, dass das neuartige Coronavirus in einem chinesischen (oder wahlweise) amerikanischen Labor entwickelt und vorsätzlich in Umlauf gebracht wurde. Nutznießer sei demnach die Pharmaindustrie, die nun Impfstoff in großen Mengen entwickeln und verkaufen könne, oder auch Bill Gates und sein angebliches Projekt der Bevölkerungsreduzierung. Andere Verschwörungserzählungen wiederum konstruieren einen Zusammenhang zwischen der 5G-Mobilfunktechnik und dem Coronavirus. So werde mit dem Coronavirus zu vertuschen versucht, dass das durch die Mobilfunktechnologie ausgelöste 5G-Syndrom die tatsächliche Ursache für den Tod zahlloser Menschen sei.

## Beispielanalysen

Heiko Schrang, über den es keinen Wikipedia-Eintrag gibt, betreibt einen eigenen Youtube-Kanal namens »SchrangTV«, der laut Focus Online 147.000 Abonnent*innen erreicht: »Seine Videos werden bis zu zwei Millionen Mal geklickt.«^17^ Schrang stellt sich selbst als bekennenden Buddhisten dar und bedient neben politischen auch esoterische Themen. Sein Kanal »SchrangTV« trägt den Untertitel: »Erkennen. Erwachen. Verändern.« Auf seiner Homepage (www.heikoschrang.de) gibt es neben Kacheln mit den Aufschriften »SchrangTV« und »Talk« auch eine mit der Aufschrift »Spirit«, hinter der sich Angebote für esoterikaffine Internetnutzer*innen finden.

**Abb. 1:**
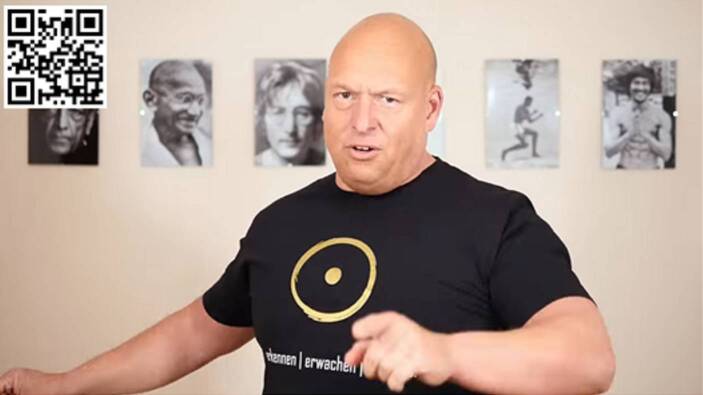
Screenshot aus dem Video »Der teuflische Plan hinter Covid-19« (01.04.2020)

In den Videos bedient Schrang sich stets des gleichen Settings: Er lässt sich in naher Einstellungsgröße (*head and shoulder close-up*) vor einer Wand filmen, an der gerahmte Fotos zu erkennen sind (u.a. von Mahatma Gandhi und John Lennon^18^). In dieser Einstellung sind Mimik und Gestik deutlich zu erkennen (Abb. 1).

Schrang trägt ein schwarzes T‑Shirt, das mit seinem Markenzeichen versehen ist, einem Kreis, in dessen Mitte sich ein großer Punkt befindet. Darunter steht der Slogan: »erkennen | erwachen | verändern«.

Für die folgende Analyse wurden sechs Videos von SchrangTV ausgewählt^19^, die zwischen Anfang März und Ende April 2020 veröffentlicht wurden und an deren Titel bereits abzulesen ist, dass sie sich mit der Corona-Krise beschäftigen:

- 02.03.2020 – Coronavirus – die totale Täuschung?
- 09.03.2020 – Corona – das Trojanische Pferd
- 23.03.2020 – Corona: Die unangenehme Wahrheit!?
- 01.04.2020 – Der teuflische Plan hinter Covid-19
- 06.04.2020 – Horror – jeder bekommt einen Impf-Chip!
- 27.04.2020 – Die Lüge hinter der Maskenpflicht.

Die Formulierung derartig marktschreierischer Titel dürfte darauf angelegt sein, den Zuschauer*innen zu signalisieren, dass hier brisante Ausführungen zu erwarten sind, die bei SchrangTV exklusiv zur Verfügung stehen. Dieser Aspekt, der von Schrang stets mit sehr deutlicher Kritik an den Qualitätsmedien und einem Aufruf verbunden wird, sich »alternativer« Medien zu bedienen, soll im Folgenden nicht vertieft werden.^20^ Vielmehr steht die Art der Argumentation im Vordergrund, die Frage, in welcher speziellen Weise Schrang Erzählungen zur Unterstützung seiner Argumentation einsetzt.

In Schrangs Videos finden sich Behauptungen, die Teil seiner Gesamtargumentation sind und mantraartig in den Corona-Videos wiederholt werden. Sie werden hier kurz aufgelistet, um den verschwörungstheoretischen Kontext zu verdeutlichen, aus dem die folgenden Beispiele stammen. Grundsätzlich stellt sich Schrang als jemand dar, der den Dingen auf den Grund geht, der nichts unhinterfragt lässt: »Wenn du das glaubst, was alle glauben, dann wird es höchste Zeit, dass du anfängst, deine Einstellung zu überdenken.« (SchrangTV, 23.03.2020; 00:12–00:20)

Die geforderte Einstellungsänderung wird nach Schrang durch die Erkenntnis gerechtfertigt, dass die von ihm als »Mainstream« bezeichneten Medien ihrer Aufgabe nicht gerecht würden und nur im Sinne der Herrschenden berichteten und »letztendlich von dieser Elite gelenkt werden« (SchrangTV, 01.04.; 4:50). Wir lebten mithin »in einer Meinungsdiktatur sondergleichen« (SchrangTV, 09.03.; 03:07), denn »es geht darum, wie die Meinungsfreiheit immer mehr außer Kraft gesetzt werden soll. Und das ist der große Plan, der dahinter steckt [...].« (SchrangTV, 09.03; 5:24–5:27)

Die Herrschenden verfolgten diverse Geheimpläne, die jedoch mehr oder weniger geschickt vertuscht würden. Mit einem dieser Pläne werde das Ziel verfolgt, das Bargeld abzuschaffen bzw. eine Weltwährung einzuführen. Aus diesem Grund werde in Zeiten des Coronavirus auch dazu aufgerufen, möglichst bargeldlos zu bezahlen. Es sei außerdem von langer Hand geplant, eine »neue Weltordnung« einzuführen. Weiterhin sei davon auszugehen, dass auch die Corona-Krise von der Regierung für ihre Zwecke missbraucht werde: »Schaut Euch genau an, was man jetzt bezwecken will. Was soll die nächste Stufe sein? Dass wir alle quasi in Quarantäne gesetzt werden, dass man jetzt über uns entscheidet, wann wir wo das Haus verlassen sollen. Denn [dann] drüber entscheidet als nächste Stufe, was wir zu denken haben? Dass wir dann gleich den RFID-Chip bekommen?« (SchrangTV, 09.03.; 17:12–17:27)

Die von Schrang erkannten Verschwörungen ließen sich aufdecken, indem man sich immer wieder – wie Schrang – »die W‑Fragen stellt: Warum ist das jetzt gerade da, wovon könnte abgelenkt werden, wer hat ein Interesse daran?« (SchrangTV, 09.03.; 7:52–7:58) Schrangs Überlegungen sind also von einem grundsätzlichen Misstrauen geprägt, das wohlwollend auch als kritisches Bewusstsein gedeutet werden mag.

### Narrative Pro-Argumente ad hominem et ad populum

Schrang beginnt seine Ausführungen meist mit einem Rückblick, in dem er darüber erzählt, was in der Zeit seit seinem letzten Podcast geschehen ist.^21^ Die argumentative Richtung dieser kurzen Rückblicke besteht darin zu verdeutlichen, dass Schrang trotz widriger Umstände dafür kämpft, der Wahrheit ans Licht zu verhelfen. Diese Wahrheit, die sich mehr oder weniger exklusiv^22^ in Schrangs Betrachtungen finde, werde von mächtigen Institutionen zu unterdrücken versucht. Doch es gelinge Schrang durch außerordentlichen Mut und Tatkraft wie auch durch die Inkaufnahme persönlicher Nachteile, seine Wahrheiten zum Wohle seiner Zuhörer*innen zu veröffentlichen: »Ich kann aber sagen, ich ertrage das für euch und dank euch. Und dank eurer Hilfe ist das überhaupt nur machbar.« (SchrangTV, 06.04.; 5:35–5:46).

Derartige Rückblicke werden narrativ eingeleitet, indem etwa von unerhörten Zensurmaßnahmen gegen Schrang erzählt wird (1, 2) oder aber indem Schrang sich unverhohlen mit Sokrates vergleicht (3).

1. Hallo und herzlich willkommen zur neuen Sendung von SchrangTV. Am letzten Freitag haben sich Dinge ereignet, die ich so noch nie in meiner Karriere erlebt habe. Alle von euch, die meinen Newsletter abonniert haben beziehungsweise in den sozialen Medien mir folgen, haben festgestellt, dass ein Sondernewsletter rauskam von mir: »Corona, Geheimplan der Regierung entdeckt«. Und was dann passiert ist, darüber reden wir jetzt einmal – für alle, die das erlebt haben und für alle, die noch nicht wissen, was überhaupt passiert ist. Also, wir haben diesen Artikel rausgeschickt, innerhalb von 2‑3 Minuten circa wurde meine Homepage gesperrt, mein Shop gesperrt, das Content-Management-Redaktionssystem gesperrt, es ist nicht aufrufbar gewesen das Video. Es war eine konstatierte [sic!] Aktion noch nie da gewesener Art. Und das ist, was ich immer sage, die wahren Verschwörer denken immer, mit ihrem Hammer schlagen sie auf das Radio drauf und die Musik ist dann weg. Funktioniert natürlich nicht, das heißt in null Komma nichts hat sich die Sache verbreitet: Heiko Schrangs Homepage wurde gesperrt, da ist irgendwas dran, was ist da los und so weiter. Wir hatten Glück, muss ich dazu sagen, ich habe noch aus alten Zeiten einen kleinen Redaktionsplatz bei Wallstreet online und, ja lesen so ungefähr 1000 bis 3000 Leute meine Artikel – also nicht erwähnenswert – und wir haben den dort auch aufgestellt. Wir blenden mal kurz den Screenshot ein: in null Komma nichts haben 150.000 Leute, unvorstellbar, diesen Artikel dort genommen, weiter geteilt und so weiter, hochgeladen, in WhatsApp-Gruppen verteilt und so weiter, also dieser Artikel wurde über eine halbe Million Mal mindestens, was ich selber nachzählen kann, gelesen. Wie viele es wirklich waren, weiß ich nicht, in WhatsApp-Gruppen und auf anderen Seiten [...]. (SchrangTV, 02.03.2020; 00:10–2:08)
2. Gegen uns werden ja Geschütze aufgefahren, wisst ihr selber, ob Bild-Zeitung, Die Zeit und wie sie alle heißen. Jede Woche kommt noch mehr, aber jetzt ist eine Sache passiert, die mir nicht gefallen hat. Und zwar mein Facebook-Account, der schon sehr, sehr lange bestanden hat, der wurde einfach vom Netz genommen. Also nicht gesperrt, einfach vom Netz genommen, und das ist natürlich ein schwerer Schlag, weil viele von euch kennen mich noch aus den Zeiten, über Facebook sind sie zu mir gekommen, wir sind immer größer geworden, wir sind eine Familiengemeinschaft kann man sagen. Menschen, die meinem Format folgen und die auch hier SchrangTV über Facebook jede Woche gesehen haben, ist jetzt vorbei. Natürlich deswegen größerer Schlag, nachdem letztes Jahr auch, wie ihr wisst, PayPal einfach gekündigt worden ist. Es ist klar, wir sind einfach mal mittlerweile, wir alle, auch ihr, die mir folgt, sind mittlerweile systemrelevant geworden mit unseren Inhalten. Ich erinnere nur an Greta beispielsweise, wo ich darüber Kurzvideo gemacht habe und auch über das Oma-Bashing, und da haben wir weit über eine Million Aufrufe dort bekommen. Es ist nur so, dass ich in diesem Fall, obwohl ich sonst nach buddhistischer Art mich meist entspanne, hier aber gegen vorgehen werde, und zwar, es ist so, dass ich die Anwaltskanzlei von Dr. Christian Stahl beauftragen werde [...] (SchrangTV, 09.03.2020; 1:01–2:18)
3. Eine Persönlichkeit, die ich mehr als schätze, ist zwar schon einige Jahre her, und zwar aus dem alten Griechenland, Sokrates, ein großer Philosoph, große Persönlichkeit, auch ein Vorbild von mir. Der Mann musste ja den Schierlingsbecher trinken. Ich meine, er hat ihn von alleine damals getrunken. Warum? Weil dieser Mann nicht nur Philosoph war, sondern der hat einfach Fragen gestellt, der hat unangenehme Fragen damals zu seiner Zeit gestellt, und man hat damals gesagt, bei dem Verfahren gegen ihn, also vor Gericht quasi, dass er die Jugend verderbe. Und was wir heute machen werden, in Zeiten, wo ich mich ganz gut mit Sokrates identifizieren kann, wo alles quasi, was irgendwie eine öffentliche, veröffentlichte Meinung infrage stellt, wird bekämpft, deswegen werden wir, weil es ein sensibles Thema ist, heute viele Fragen stellen. (SchrangTV, 23.03.2020; 1:03–2:01)

Die Funktion dieser narrativen Elemente ist in erster Linie in der positiven Selbstdarstellung Schrangs bei gleichzeitiger Abwertung seiner Kontrahenten zu sehen. Er stilisiert sich in diesen Erzählungen als unerschrockener Wahrheitssucher, der auch vor persönlichen Nachteilen nicht zurückschreckt. Obwohl er von den tatsächlichen Verschwörern mit allen Mitteln massiv bekämpft wird, gibt er nicht auf. Die Zuhörer*innen müssen Schrang nur aufmerksam folgen, um so von seiner selbstlosen Tätigkeit zu profitieren und an seinem Erkenntnisgewinn teilhaben zu können. Sie werden auf diese Weise in die Lage versetzt, die zahlreichen Ungereimtheiten zu durchschauen, die Schrang für sie als das erwiesen hat, was sie tatsächlich sind: eine Verschwörung von oben, die sich gegen »Menschen wie du und ich«, gegen das gemeine Volk richtet.

Die Funktion der exemplarisch zitierten Narrationen besteht mithin darin, Schrang als jemanden erscheinen zu lassen, der stellvertretend für seine Rezipient*innen verborgene Wahrheiten aufdeckt und sodann sein exklusives Wissen über Verschwörungen den nichtwissenden Massen mitteilt. Ein dermaßen selbstloses Handeln, wie Schrang es an den Tag lege, könne nur durch altruistische Motive erklärt werden. Diese werden in den Narrationen Schrangs fokussiert. Sie fungieren als moralische Legitimation der im Anschluss erfolgenden Argumentation. Als Fazit könnten Schrangs Rezipient*innen daraus ziehen: Jemand, der so selbstlos und unermüdlich wie Schrang gegen die Mächtigen agiert, dem muss man Glauben schenken, der ist über kleinliche Zweifel erhaben.

Eine weitere Funktion der einleitenden Narrationen ist darin zu sehen, die Existenz der von Schrang behaupteten Verschwörung(en) zu plausibilisieren. Werde jemand wie Schrang, der altruistisch im Dienste der Wahrheit und der Demokratie arbeite, dermaßen mit unfairen und rechtswidrigen Mitteln an seiner Arbeit gehindert, dann sei davon auszugehen, dass eine Verschwörung gegen ihn im Gange sei. Diese Verschwörung richte sich allerdings nicht ausschließlich gegen Schrang, da dieser ja stellvertretend für seine Rezipient*innen – und alle, die es noch werden wollen – tätig sei. Es dürfte sich folglich um eine groß angelegte Verschwörung der politischen Elite gegen das Volk handeln.

### Narrative Analogie-Argumente

Manfred Kienpointner hat in seinen Untersuchungen zur »Alltagslogik« (1992, S. 384–393) gezeigt, wie Beispiele und Analogien Argumentationsmuster liefern, die zur Plausibilisierung einer Argumentation beitragen können.^23^ Analogien sind dadurch gekennzeichnet, dass sie unterschiedliche Realitätsbereiche in Beziehung setzen und auf diese Weise Gemeinsamkeiten oder Unterschiede zwischen diesen Bereichen verdeutlichen. Sie können insbesondere auch der Didaktisierung dienen, »wenn der Argumentierende davon ausgehen muß, daß sein Gegenüber andere oder geringere Wissensvoraussetzungen (unterschiedliche oder teilweise nicht vorhandene enzyklopädische Kenntnisse) hat« (Kienpointer 1992, S. 387). Da Schrang mit der Veröffentlichung seiner Videos vorauszusetzen scheint, dass sie einen Neuigkeitswert für seine Rezipient*innen haben, er mithin einen Wissensvorsprung vor seinen Rezipient*innen habe, sind Analogien bestens geeignet, den Zuschauer*innen in paternalistischer und gleichzeitig missionarischer Weise seine Sicht der Dinge zu erklären. Auch bei derartigen Argumentationsmustern bedient Schrang sich immer wieder narrativer Elemente.

(4). [...] und so motiviert wie das jetzt klingt, hat natürlich die ganze Sache auch eine dunkle, eine Kehrseite, weil, ihr müsst euch das so vorstellen: Heiko Schrang – dankeschön, dass ihr mir folgt seit Jahren – der sitzt in seinem Paddelboot auf dem Meer und mittlerweile ist es so, von Woche zu Woche, dass die Kriegsschiffe immer größer werden, die aufgefahren werden, also quasi die Wellen gegen mein Boot und die Schüsse, die kommen, die werden immer größer. Das heißt, unser kleines Paddelbötchen das kommt massiv ins Wanken, und deswegen ist es jetzt an der Zeit – viele von euch haben natürlich mich auch unterstützt, finanziell unterstützt, dass wir uns diese Paddel kaufen konnten, und ab und zu haben wir einen neuen Anstrich dran gemacht, der konserviert das Boot. Aber jetzt ist der Punkt da, wo wir uns ein Schnellboot kaufen müssen quasi, ein Schnellboot, was wendig ist, um all diesen Angriffen, die ja massiv zunehmen, auszuweichen. Und deswegen an dieser Stelle nochmals vielen Dank an alle Unterstützer von euch und auch an alle zukünftigen, die überhaupt meine Arbeit ermöglichen. Ist mir wirklich wichtig, das an der Stelle mal gesagt zu haben. Und auch für alle, die noch nicht den Newsletter abonniert haben, den endlich zu abonnieren, weil am Freitag, was dort passiert ist, zum einen war es eine Riesen-Anteilnahme von eurer Seite und eine Riesenhilfe, dass ihr das überall verbreitet habt, das hat uns gezeigt, wie groß wir sind, dass wir eine riesengroße Gemeinschaft sind, die wirklich systemrelevant ist, also nicht eine kleine Pille-palle-Truppe, die man so wegschieben kann. Das ist der Grund, warum die Bild-Zeitung und noch andere alle großen Geschütze gegen meine Person auffährt. (SchrangTV, 02.03.2020; 8:59–10:31)
(5) Diese öffentlich-rechtlichen Medien sind für mich vergleichbar wie so ein riesengroßes Fischernetz im riesengroßen Atlantik oder Pazifik, ist egal, und dort drin sind die ganzen Fische, ganz eng beieinander, Schulter an Schulter. Und die Fische, das ist die Masse der Bevölkerung. Die haben in diesem Netz dringehangen und man hat ihnen immer erklärt: »Ist doch schön kuschelig, ist doch toll! Wir sind doch für euch da, ihr müsst aber dies und das tun!«, quasi sinnbildlich, für uns Steuern zahlen und so weiter. Und dann gab es zwei, drei Fische, das waren die, die auch ein bisschen gelesen haben, aber Dinge, Bücher gelesen haben, die abseits des Mainstreams waren. Die sind aber unwichtig gewesen für dieses riesengroße Netz, die sind da irgendwo rumgeschwommen im Atlantik. Und auf einmal kamen Typen wie Heiko Schrang, andere Berufskollegen von mir, die außerhalb des Netzes unterwegs waren, und die sagten: »Hallo Fischlein, wenn ihr wollt, braucht ihr gar nich im Netz drin sein. Ihr könnt auch freischwimmen, kommt doch einfach mit mit uns. Jeder kann freischwimmen im Ozean!« Und dann gab es ganz viele, und die nehmen mittlerweile von Tag zu Tag – nicht von Woche zu Woche – immer mehr zu, gerade in diesen Corona-Wahnsinns-Zeiten, die dieses große Netz verlassen. Dieses große Netz bekommt da ’n Loch, da noch ’n Loch, da noch ’n Loch und schwimmen letztendlich uns hinterher. Und davor haben die richtig Angst, aber richtig Angst, und im Endeffekt sagen wir: »Jeder kann hinschwimmen, wo er will.« Aber die Leute beziehungsweise der Besitzer des Netzes oder die, die dieses Netz quasi in ihren Händen halten, die sagen natürlich: »Wenn ihr da rausschwimmt, wenn ihr da hinschwimmt, da herrscht große Gefahr für euch, da könnt ihr sterben! Und jetzt grade könnte es sogar sein, dass ihr irgendwelche bösen Bakterien oder Viren bekommt. Und das sind nur Scharlatane, das sind nur Lügner, die da draußen schwimmen. Da ist ja gar keine Freiheit, das stimmt ja gar nicht, das ist ja quasi eine Illusion, also irgend so eine Art virtuelle Welt, die die euch nur eingeben. Es stimmt, die sind ja gar nicht frei.« Und das erzählt man denen, die quasi grade aus dem Netz herauswollen, die noch zurückgucken und sagen: »Vielleicht haben die ja Recht.« Aber die Mehrzahl bleibt nicht mehr, die geht jetzt raus. So, und die sind irgendwann draußen und drehen sich zurück und sagen: »Was habe ich denn eigentlich gemacht? Jahrzehntelang gefangen in diesem Netz!« Und die Beweise, liebe Leute, sind ganz simpel, ganz simpel! (SchrangTV, 01.04.2020; 5:30–7:57)
(6) Das müsst ihr euch so vorstellen, jeden Tag wird jetzt ein Steinchen, kommt einer vom Steinchen hochgekrochen und die sagen sich: »So, es hat nicht funktioniert, du, schmeiß mal jetzt ’ne Bananenschale, vielleicht rutscht er ja aus.« Hat nur nicht funktioniert. Dann kommt der nächste, der kippt dann Öl aus, dass wir irgendwie ausrutschen drauf. Das funktioniert irgendwie alles nicht. Also, Jungs und Mädels, das könnt ihr euch echt sparen. Es wird langweilig auch mittlerweile. (SchrangTV, 01.04.2020; 13:02–13:27)

Die Funktion derartiger narrativ entfalteter Analogien, die wie Beispiel (5) auch Parabel-Charakter haben können^24^, ist in der Stützung von Schrangs Argumentation zu sehen, indem den Zuhörer*innen Situationen vor Augen geführt werden, die sie entweder sehr leicht entschlüsseln können, weil die Symbolik überdeutlich ist (Paddelboot gegen Kriegsschiffe, im Netz gefangene/freie Fische) oder aber Alltagswissen abruft (Bananenschale, Ölspur als gefährliche Stolperfalle bzw. Unfallursache). Bei derartigen Analogien wird häufig auch noch ein David-gegen-Goliath-Frame aufgerufen und somit sowohl an das Mitleids- wie auch das Gerechtigkeitsempfinden der Zuhörer*innen appelliert. Schließlich werden die Anhänger*innen Schrangs als Gruppe bzw. Familie angesprochen, um das Gemeinschaftsgefühl anzusprechen. Im Zusammenspiel mit der Selbstpositionierung Schrangs als Wahrheitssucher, der von der herrschenden Elite systematisch bekämpft wird, ergibt sich ein Appell an die Solidarität der Zuhörer*innen, die ja allesamt von Schrangs aufopferungsvoller Arbeit profitieren.

### Narrative Contra-Argumente ad hominem

Gelingt es, Widersprüche in gegnerischen Argumentationen aufzuzeigen, so hat man die Logik auf seiner Seite und kann die erhobenen Geltungsansprüche der gegnerischen Seite delegitimieren. Schrang weist in seinen Videos häufig auf Widersprüche hin, die er mittels narrativer Elemente in Szene setzt. So galten zum Zeitpunkt der von Schrang in Beispiel (8) geschilderten Beobachtungen offensichtlich unterschiedliche Richtlinien zum Tragen von Masken in der Stadt Berlin und im direkt angrenzenden Bundesland Brandenburg.

Allerdings fällt auf, dass die von Schrang diagnostizierten Widersprüche häufig nur dadurch entstehen, dass Argumente einer Person (z. B. eines Experten) aus verschiedenen Zeiträumen als nicht konsistent deklariert werden bzw. aufgrund des zeitlichen Abstands nicht konsistent sind oder dass Positionen bzw. Verhaltensweisen verschiedener Menschen oder Gruppierungen gegenübergestellt werden. So gab es im Verlaufe des Monats März 2020 beispielsweise sich verändernde Positionen in Bezug auf die sogenannte Maskenpflicht, die schließlich Ende April 2020 in allen Bundesländern eingeführt wurde. Der in Beispiel (9) gezeigte Widerspruch zwischen der Propagierung einer Maskenpflicht und dem Verhalten von Journalist*innen beruht auf der Gleichsetzung der von Schrang sogenannten Mainstream-Medien, die die Bürger*innen zum Tragen von Atemschutzmasken aufrufen, und einer Gruppe maskenloser Journalist*innen, die Annegret Kramp-Karrenbauer bei der symbolträchtigen Übergabe einer großen Charge derartiger Masken interviewen wollen. Als widersprüchlich wird ebenfalls die Einreise von Erntehelfern angesichts der generellen Reisewarnung dargestellt.

(7) Wir stehen kurz vor Ostern. Ihr dürft jetzt nicht – als Beispiel – an die Ostsee fahren. Gibt Leute, die haben dort ein Zweitwohnsitz, die haben da ’ne Ferienwohnung, dort darf man nicht hin, die dürfen dort nicht hin! Ja, aber 80.000 Erntehelfer kommen jetzt nach Deutschland. Also wenn es so schlimm wäre, stellt sich die Frage: Warum eigentlich, warum kommen die jetzt hierher? (SchrangTV, 06.04.2020; 7:30–7:52)
(8) Und das sind natürlich Sachen, wo man sich mal die Frage stellen soll: Was läuft hier ab? Und grade bei uns, ich komme ja aus Brandenburg, da ist mir heute eins aufgefallen: Da besuche ich einen Bekannten in Glienicke/Nordbahn, das ist im Norden von Berlin und – im Norden von Brandenburg –, und kurz dahinter kommt Frohnau, ein Bezirk der Schönen und Reichen. Und dort sehe ich im Supermarkt, wie sie alle wie die Wahnsinnigen mit Masken rumrennen, im Vorbeifahren schon. Ich dachte: Sind das alles Bankräuber, was machen die denn alle da? Rauben die jetzt den Laden aus? So sah das aus, völlig irreal die ganze Szenerie, und 100 Meter weiter da hab’ ich mir gleich gesagt: »Heiko! In Berlin ist noch keine Maskenpflicht, alles in Ordnung.« 100 Meter weiter der nächste Supermarkt in Berlin. Da habe ich mir so die Frage gestellt: Was ist denn da eigentlich los? Hat der Virus letztendlich ein Abkommen mit Erich Honecker damals gemacht, war das quasi letzte Rache von Erich, so nach dem Motto: »Oh nee, also ihr habt jetzt unser Volk verraten, weil ihr seid ja zu den bösen Leuten aus der BRD rübergegangen, also werde ich es mal so konzipieren, dass der Virus, der nur im Ostteil bleibt, im ehemaligen Ostteil, also der geht nicht rüber, also eine imaginäre Grenze ist da anscheinend gezogen worden.« (SchrangTV, 27.04.2020; 6:49–8:02)
(9) Schaut euch bitte mal das andere Bild an! Ich wusste wirklich nicht, ob ich lachen oder ob ich heulen sollte. Die Polit-Darstellerin Kramp-Karrenbauer, die ja die Nachfolge übernommen hat von Flintenuschi, Ursula von der Leyen, mit ihrer Kompetenz, ja kann ich jetzt nicht sagen, aber im Endeffekt sind es immer die gleichen Personen, die in so eine Position kommen. Fakt ist aber eins: Guckt euch mal an, wie Journalisten, die übereinander herfallen, fast kuscheln, wo ich dachte: Oh, gibt es jetzt einen Hals-Nasen-Ohren-Kuss noch, weil ihr so nah aufeinander alle hockt? Die fallen übereinander, schlecken sich fast alle ab, aber, wichtig: Wir haben aber Maskenpflicht! Heißt, die Masken kommen grad hier an, aber hamse noch nicht geschafft auszupacken für sich selber die Maske. (SchrangTV, 27.04.2020; 6:01–6:48)

Durch die narrativen Elemente im Beispiel (8) werden die (absurden) Folgen szenisch dargestellt, die sich ergeben, wenn man die in der gegnerischen Argumentation propagierten Maßnahmen befolgt. Diese Szenen laufen vor dem geistigen Auge der Zuhörer*innen ab und machen ihre Absurdität konkret. Eine solche Konkretheit kann durch eine abstrakte Argumentation, mittels derer auf Widersprüche hingewiesen wird, schwerlich erreicht werden. Dies gilt für die erzählten Supermarkt-Szenen, deren Absurdität ja darin besteht, dass mit dem Übertreten der Grenze eines Bundeslandes andere Verordnungen in Bezug auf die Maskenpflicht galten, während sich das Virus ungehindert über Ländergrenzen hinweg ausbreiten konnte.

Im folgenden Beispiel (9) hingegen wird bildlich und somit »auf den ersten Blick« dargestellt, welch widersprüchliches Verhalten der politische Gegner an den Tag legt: Die »Mainstream«Journalist*innen berichten über die Lieferung von Masken, die der Bevölkerung als lästiges, aber unverzichtbares Mittel im Kampf gegen die Pandemie angepriesen werden, scheinen diesen Kampf selbst aber nicht ernst zu nehmen und es daher nicht für nötig zu erachten, Masken zu tragen und die Abstandsregeln einzuhalten.

Mit der narrativen Darstellung der sich ergebenden Absurditäten und Widersprüche ist bei Schrang jedoch noch eine weitere argumentative Funktion verbunden. Sie besteht darin, die von ihm – sozusagen als Grundrauschen – verbreitete These von einer großen Verschwörung der Mächtigen gegen das Volk zu plausibilisieren: Wenn es zu solchen wie den geschilderten Absurditäten kommt, kann man die Argumentation des Gegners nicht ernst nehmen – sie führt sich vielmehr selbst ad absurdum. Es handelt sich mithin nicht um eine wahrhaftige Argumentation, sondern um ein Vertuschungsmanöver, das von den wahren Motiven der Handelnden ablenken soll. Es liegt also nahe, von einem Geheimplan bzw. einer groß angelegten Verschwörung auszugehen.

### Narrative Argumente von der Handlung auf die Intention

Verschwörungstheorien – so wurde John D. Seidler (2016) anfangs zitiert – beziehen sich stets auf ein bestehendes Narrativ, dessen Fehlerhaftigkeit sie durch ein konträres Narrativ aufzudecken suchen. Deshalb ist es nicht verwunderlich, dass die Argumentation von Verschwörungstheoretikern zu großen Teilen Contra-Argumentation ist, die ein Narrativ und die mit ihm als gültig vorausgesetzten Argumente als widersprüchlich, nicht plausibel etc. darstellen will. Dies funktioniert in den Videos von Schrang in erster Linie dadurch, dass er Generalisierungen vornimmt, die es ihm sehr leicht machen, Widersprüche aufzuzeigen. So wird etwa ignoriert, dass wissenschaftlicher Fortschritt in erster Linie durch Widerspruch zustande kommt, dass wissenschaftliche Hypothesen – zumindest idealtypisch – immer auch als Einladung zu einer kontroversen Diskussion gemeint sind. Wird diese Tatsache systematisch ausgeblendet, dann fällt es leicht »den Experten« oder »der Wissenschaft« Glaubwürdigkeit abzusprechen, weil sie sich ja »nicht einig« sei(en) oder sich gar »widersprechen«. Ähnliches gilt für »die Politik« bzw. »die Politiker«. Wird negiert, dass auch eine Demokratie vom Widerspruch lebt, und wird darüber hinaus Politiker*innen nicht zugestanden, Einsichten hinzuzugewinnen, aufgrund derer sich die eigene Position ändern kann, dann fällt es nicht schwer, auch in diesem Bereich Widersprüche aufzuzeigen und anzuprangern. Während der Corona-Krise ließen sich sowohl auf Seiten der Wissenschaft, insbesondere der Virologie, wie auch auf Seiten der Politik solchermaßen konstruierte Widersprüche aufzeigen: Diese betrafen etwa Sinn und Zweck einer Maskenpflicht oder die Bedeutung verschiedener Maßzahlen (Reproduktionszahl vs. Zahl der Neuinfektionen). Auf der politischen Agenda standen insbesondere Maßnahmen zur Eindämmung des Coronavirus wie Ausgangsbeschränkungen, Versammlungsverbote und später die sogenannten Lockerungen, die – entsprechend der föderalen Struktur der Bundesrepublik Deutschland – in den einzelnen Bundesländern unterschiedlich gehandhabt wurden. Dass die Corona-Krise tatsächlich auch für Politiker*innen eine Ausnahmesituation darstellt, macht Gesundheitsminister Jens Spahn in der Parlamentsdebatte am 22. April 2020 deutlich, wenn er selbst auf Widersprüche hinweist, die der Tatsache geschuldet seien, dass sich der Wissensstand über die Bekämpfung des Virus mit jedem Tag verändere:

Bei etwas anderem bin ich ausdrücklich Ihrer Meinung – das will ich auch grundsätzlich zu anderen Debatten, etwa auch gerade zur Maske und anderem, sagen –, dass wir nämlich miteinander in ein paar Monaten wahrscheinlich viel werden verzeihen müssen, weil noch nie [...] in der Geschichte der Bundesrepublik und vielleicht auch darüber hinaus in so kurzer Zeit unter solchen Umständen mit dem Wissen, das verfügbar ist, und mit all den Unwägbarkeiten, die da sind, so tiefgreifende Entscheidungen haben getroffen werden müssen; das hat es so noch nicht gegeben. Ich bin immer ganz neidisch auf diejenigen, die schon immer alles gewusst haben.

[...] Ich bin mir sicher: Jenseits von Politik wird auch für die Gesellschaft, selbst für Virologen und Wissenschaftler, eine Phase kommen, wo wir alle im Nachhinein feststellen werden, dass man vielleicht an der einen oder anderen Stelle falschgelegen hat oder an der einen oder anderen Stelle Dinge dann noch mal korrigieren und nachsteuern muss. [...] (Plenarprotokoll Deutscher Bundestag 19/155 v. 22.04.2020, S. 19211 [B])

Auf Widersprüche in der Argumentation oder im Handeln anderer hinzuweisen, macht allerdings noch keine Verschwörungstheorie bzw. -erzählung aus. Verschwörungserzählungen geben zusätzlich stets Antwort auf die Frage, worin der Plan der Gegenseite, mithin die Verschwörung besteht, und sie liefern Argumente für das Vorhandensein einer Verschwörung. In diesem Zusammenhang tauchen bei Schrang stets wiederkehrende Argumente auf, die mehr oder weniger plausibel mit der Corona-Krise in Zusammenhang gebracht werden. Dies erfolgt in der von Butter (2018, S. 106) beschriebenen Weise: »Wo andere Chaos oder Dummheit sehen, erkennen sie [die Verschwörungstheorien, Th. N.] Muster und Intentionen. Es macht einen großen Teil ihrer Attraktivität aus, dass sie Disparates verbinden, dass sie statt auf Zufall und Kontingenz auf Kohärenz und dunkle Absichten setzen.« So bedienen sie das zutiefst menschliche »Bedürfnis, Ereignisse als das Resultat intentionaler Handlungen zu begreifen« (Butter 2018, S. 107).^25^

Die in diesem Zitat angesprochenen Intentionen der Verschwörer werden in Schrangs Videos immer wieder thematisiert. Sie umfassen verschiedene Bereiche und lassen sich unter dem Aspekt zusammenfassen, dass die Verschwörer – mithin »die Politik« – die Rechte der Bürger*innen und sogar Staatsrechte massiv einschränken wollen. Insbesondere folgende Rechte sind laut Schrang betroffen:

- das Recht, mit Bargeld zu bezahlen, sowie das Recht auf eine nationale Währung,
- das Recht auf körperliche Unversehrtheit (angeblich geplante Impfpflicht)^26^,
- das Recht auf staatliche Souveränität (Migration und »neue Weltordnung«^27^),
- das Recht auf Meinungsfreiheit (angebliche Zensurmaßnahmen).

Die Intentionen der Verschwörer, diese Rechte einzuschränken bzw. abzuschaffen, stellen die Prämissen von Schrangs Argumentation dar, die als gesetzt gelten. Das zugrunde liegende spezifische Schlussverfahren besteht darin, ausschließlich nach solchen Indizien zu suchen, die diese Prämissen stützen könnten. Es lässt sich am Bargeld-Beispiel wie folgt rekonstruieren:

[Die Politik will das Bargeld abschaffen.]^28^

Wenn die Politik das Bargeld abschaffen will, wird der erste Schritt darin bestehen, die Bürger*innen zu bargeldlosem Zahlen aufzurufen.

Während der Corona-Krise werden die Bürger*innen zunehmend dazu aufgerufen, bargeldlos zu zahlen.

Die Politik will also das Bargeld abschaffen, und die Corona-Krise ist nur ein Vorwand, die Abschaffung des Bargelds durchzusetzen.

Diese Art zu argumentieren, scheint ein Charakteristikum von Verschwörungserzählungen zu sein. Michael Butter (2018, S. 59) bringt dies auf die treffende Formel, dass Verschwörungstheoretiker »Geschichte immer vom Ende her [erzählen]«:

Wenn sie mit ihrer Untersuchung beginnen, wissen Verschwörungstheoretiker immer bereits, wer die Schuldigen sind. Entsprechend ist ihre gesamte Beweisführung darauf ausgerichtet, ihren Verdacht zu bestätigen. [...] Die anfangs als schuldig Identifizierten bleiben dies in aller Regel auch, es können lediglich weitere Schuldige hinzukommen. [...] Der Konspirationismus löst eine vielschichtige und widersprüchliche Wirklichkeit in den manichäischen Gegensatz von Gut und Böse auf. Der meist kleinen Gruppe von Verschwörern [...] steht die große Gruppe der Opfer gegenüber, die bis auf wenige Erleuchtete gar nicht begreift, was passiert. (Butter 2018, S. 60)

Wie die Plausibilität derartiger verschwörungstheoretischer Schlussfolgerungen beurteilt wird, hängt ganz entscheidend von der politischen Grundeinstellung eines Menschen ab sowie von seinem Vertrauen in staatliche Organe bzw. die jeweilige politische Führung. Schrang entfaltet die Prämissen seiner konklusiven Sprachhandlungen immer wieder narrativ, um durch die Anknüpfung an alltagsweltliche Erfahrungen ihre Plausibilität zu erhöhen und für weitergehende Schlussfolgerungen anschlussfähig zu machen:

(10) Achtet bitte in den nächsten Wochen darauf, was man euch erzählt. Da wird man euch sicherlich erzählen: »Wir haben jetzt den Überträger, den bösartigen Überträger, den haben wir jetzt identifiziert. Es ist – das Bargeld. Und deswegen, damit wir nicht nochmal so eine böse Krise haben, müssen wir leider das Bargeld abschaffen.« Und wenn wir das Bargeld abschaffen, kein Problem, dann können wir machen, was wir wollen, mit euch. Das heißt, die Negativzinsen, die momentan ja bei den Banken vorhanden sind, da könnt ihr momentan ja noch hinrennen, euer Geld runterholen, unter ein Kopfkissen legen, ist nicht mehr machbar, wird einfach abgebucht, Punkt. Und wenn ihr nicht mitspielt, dann wird’s Konto zugemacht, dann könnt ihr euch nichts mehr kaufen, nichts mehr essen. Das ist der lange Plan, der schon immer da war. Neue Weltordnung – NWO! (SchrangTV, 23.03.2020; 22:52–23:39)
(11) China sagt: ›Okay, das Bargeld könnte ja infiziert sein, also müssen wir erstmal Bargeld einziehen‹. Das ist also der absolut legendäre Plan, den die teuflischen Eliten ja schon seit Jahren planen, uns das Bargeld wegzunehmen, damit wir richtig schön kontrolliert werden können. Ein viel größerer Plan steckt dahinter, und zwar der Plan, das jetzt – Chaos zu schaffen, um dann ihre Ordnung zu installieren, das alte Freimaurer-Prinzip. Deswegen wird wöchentlich alles aufgefahren, über Hanau^29^ redet gar keiner mehr, jetzt ist das das Thema. Und es war schon immer so in Kriegszeiten, in diesen Zeiten gibt es Notstandsverordnung oder Kriegsrecht, und diese machen eins: alle anderen, das normale regelrechte Recht, wo wir uns dran festhalten, gibt es dann nicht mehr. Und genau da soll die Reise hingehen. (SchrangTV, 02.03.2020; 15:21–16:07)

Für Schrang ergibt sich aus alledem die Notwendigkeit, nicht nur zur Wachsamkeit, sondern auch zum zivilen Ungehorsam aufzurufen. Dieser Aufruf wird mit narrativen Elementen untermalt, mit historischen Erzählungen untermauert, die Analogien zur DDR-Zeit wie zu biblischen Motiven konstruieren und Schrangs Argumentation stützen sollen. In der Erzählung, die auf die DDR-Zeit referiert, vergleicht sich Schrang mit den sogenannten Bürgerrechtler*innen der DDR (»diese Leute wie wir«). Diese Analogie legt die Inferenz nahe, dass Schrang zwar in den Medien als Verschwörungstheoretiker, Nazi, Antisemit etc. beschimpft werde, tatsächlich jedoch wie die erwähnten Bürgerrechtler*innen im Namen der Freiheit gegen ein Unrechtsregime kämpfe. Mit dem Bezug auf Jesus und seine Jünger wird eine Analogie zwischen Schrang nebst Rezipient*innen sowie Jesus und seinen Jüngern (bzw. Buddha und seinen Anhängern) gebildet. Neben dieser offen kommunizierten Analogie wird die implizitere Analogie zwischen Jesus bzw. Buddha und Schrang durch den Hinweis, es handele sich um eine Metapher, relativiert. Deutlich wird aber auch hier die Argumentation, die durch Analogien gestützt werden soll: Schrangs Rezipient*innen sollen sich nicht irre machen lassen und Mut zum zivilen Ungehorsam haben. Dieser werde sich trotz aller Anfeindungen im Endeffekt als segensreich für die Gesellschaft erweisen.

(12) Das ist der Stand der Dinge, den wir gerade haben. Und dann noch was, wo die Reise hingeht. Zu DDR-Zeiten hat man Leute wie Vera Lengsfeld oder Bärbel Bohley oder in den 70er-Jahren damals noch Wolf Biermann, Havemann, wie sie hießen. Das waren Bürgerrechtler. Ja, da hat man damals in dem sogenannten Westfernsehen, dem ZDF, gesprochen von Bürgerrechtlern, Freiheitskämpfer, die für die Freiheit gekämpft haben, Aktivisten, Friedensaktivisten. Und mittlerweile nennt man diese Leute wie wir Verschwörungstheoretiker, Rechtsradikale, Nazis, Antisemiten. Und in die Richtung geht es jetzt, dass der Mainstream, die gleichgeschaltete Presse, genau das schreibt über die Mitglieder der Demos, die unterwegs sind. Lasst euch nichts sagen, hört auf euer Herz. Und ich kann euch nur eins sagen: Wir sind noch mehr. Aber die Schmerzgrenze ist anscheinend noch nicht ganz da bei den Leuten, weil erst dann werden sie es verstehen. Deswegen seid ihr gefragt, dass ihr im Kleinen weiterhin mit euren Freunden und Bekannten redet. Und die völlig gegen die Wand gerannt sind, bitte loslassen, sag ich jedes Mal, weiter, der Nächste bitte, der Nächste. Ihr seid diejenigen – bei Jesus hat man damals gesagt, das waren seine Jünger. Als Metapher mein ich damit, dass Jesus gesagt hat, er hat eine Lehre gehabt, wie Buddha eine Lehre hat und so weiter, aber wenn die Leute nicht die gewesen, die die anderen, beim Erwachen, so mitgemacht hätten, dann wäre auch nichts dabei daraus geworden, dann hätten wir nicht die großen Religionen in der Welt gehabt. Und deswegen ist jeder einzelne wichtig, und das gilt für alles: Der zivile Ungehorsam und die Zivilcourage ist wichtiger denn je. Und denkt bei allen immer daran: Nur wer gegen den Strom schwimmt, der gelangt zur Quelle. Denn nur tote Fische schwimmen mit dem Strom. (SchrangTV, 27.04.2020; 19:22–21:22)

## Fazit

Am Beispiel ausgewählter Youtube-Videos des Verschwörungstheoretikers Heiko Schrang wurde versucht, mögliche Funktionen narrativer Elemente in Argumentationen zu illustrieren. Dabei zeigte sich, dass die vorkommenden narrativen Elemente den Rezipient*innen auf vielfältige Weise das Verständnis und den Nachvollzug der verschwörungstheoretischen Argumentationen Schrangs erleichtern können: »Narratives [...] provide flesh and blood for the bones of an argument, transforming both into a powerful tool of argumentation« (Tindale 2017, S. 29). Dies gilt auch für die in die Gesamtargumentationen Schrangs eingebetteten Erzählungen. Sie dienen indirekt der Stützung einer Argumentation bzw. Schlussregel im Sinne Toulmins (1996, S. 88–98, 155–156). Ihre Funktion ist darin zu sehen, Anknüpfungsmöglichkeiten zwischen dem Wissen der Rezipient*innen und Schrangs Verschwörungserzählungen zu schaffen. Diese werden durch narrative Elemente plausibilisiert, die mit dem Alltagswissen der Rezipient*innen vereinbar sind. Sie werden durch narrative Analogien konkretisiert, die den Rezipient*innen ebenfalls aus ihrem Alltagswissen vertraut sind. Schließlich wird mittels des Rückgriffs auf historisches Wissen an das kollektive Gedächtnis bzw. Gewissen appelliert, um die Notwendigkeit zivilen Ungehorsams in der heutigen Zeit zu begründen. Sowohl die Legitimierung des eigenen wie die Delegitimierung gegnerischer Standpunkte erfolgt dabei *ad hominem*, d.h. unter Vernachlässigung von Sachargumenten. Stattdessen werden Behauptungen über die Intentionen und Motive der ›Herrschenden‹ und der ›Mainstream-Medien‹ lanciert. Die Verschränkung von narrativen Elementen und Argumentation ist dabei eine doppelte: Schrangs Verschwörungserzählungen sollen argumentativ abgesichert werden und die argumentativen Absicherungen dieser Verschwörungserzählungen bedienen sich ihrerseits wiederum narrativer Elemente.

Zusammenfassend lässt sich feststellen, dass – wie bereits bei Quintilian ausgeführt – die Funktion der Narration in den untersuchten Youtube-Videos darin besteht, die Rezipient*innen auf die Sicht des Redners einzustimmen, indem Problemverhalte so dargestellt werden »wie es uns zustattenkommt« (Quintilian 2015, IV, 2, 20). Wird die perspektivische Sichtweise Schrangs von seinen Rezipient*innen übernommen, so bedeutet dies gleichzeitig, dass sie von ihnen als »objektiv« ratifiziert wird. Ein auf solche Weise erfolgreiches Framing stellt nicht nur für verschwörungstheoretische Argumentationen wie sie von Schrang präsentiert werden eine perfekte Ausgangslage dar.

## Footnotes

1 Ein kurzer Abriss zur einschlägigen Forschung in der Kommunikationswissenschaft, Psychologie, Rhetorikforschung und Linguistik findet sich bei Till (2019). Die Themenhefte »Narration – Persuasion – Argumentation« der *Zeitschrift für Literaturwissenschaft und Linguistik* (Bleumer/Hannken-Illjes/Till 2019a) sowie »Alltagspraktiken des Erzählens« (Spieß/Tophinke 2018) und die Beiträge im Sammelband von Olmos (2017) geben ebenfalls wichtige Hinweise. Dass der Ausdruck *Narrativ* seit einigen Jahren zum bildungssprachlichen Modewort geworden ist, macht Zifonun (2017) in ihrer Glosse zum Thema.

2 Vgl. zu diesem Ausdruck Stötzel (1977, S. 71).

3 Vgl. die kritische Bestandsaufnahme zu Frames und Framing bei Klein (2018).

4 Das literarische Genre, in dem der Zusammenhang von Argumentation und Rechtsanspruch ebenfalls deutlich wird, ist – nicht von ungefähr – der Kriminalroman bzw. -film. Typischerweise werden den Leser- bzw. Zuschauer*innen hier in narrativer Form vermeintliche Tathergänge aus unterschiedlichen Perspektiven präsentiert, um die Schuld bzw. Unschuld diverser Protagonist*innen argumentativ abzusichern. Die sogenannte Lösung eines Kriminalfalls ist unter argumentationsanalytischer Perspektive mithin nichts anderes als die möglichst widerspruchsfreie und überdies psychologisch plausible narrativ-argumentative Rekonstruktion eines Tathergangs und der Motive der Handelnden. Mustergültig findet sich dieses rekonstruktive Verfahren in dem Roman *Das Urteil* von Paul Hengge (1998), in dem verschiedene Zeugenaussagen zu einem Mordfall im Zentrum stehen. Die beiden Protagonisten des Romans versuchen, mittels scharfsinniger Argumentationen ihr jeweiliges Gegenüber davon zu überzeugen, dass nur sie die Motive möglicher Täter und den daraus resultierenden Tathergang korrekt einschätzen und wiedergeben. Deshalb könne auch nur ihre Version des (nicht in Zweifel stehenden) Verbrechens den Tatsachen entsprechen.

5 Richter (2008) betont in seiner Dissertation zum narrativen Urteil den Gegensatz von argumentativen und narrativen Problemverhandlungen. Im Folgenden wird dagegen eine Sichtweise vertreten, die die *narratio* als integralen Bestandteil einer im weiten Sinne verstandenen *argumentatio* auffasst; vgl. dazu auch Tindale (2017, S. 12–25), der ebenfalls eine weite bzw. »dynamische« Auffassung von Argumentation vertritt.

6 Darauf weist bereits Dieckmann (1964, S. 71 f.) in seiner Dissertation hin, in deren Untertitel der Ausdruck *politische Werbung* prominent platziert ist.

7 Vgl. dazu ausführlicher Vogel (2018).

8 Zur Semantik von *Schlagwort* und *Leerformel* vgl. Niehr (2019a, S. 678 f.).

9 Die folgenden Überlegungen fußen auf den grundlegenden Publikationen von Butter (2018) und Seidler (2016).

10 Vgl. dazu ausführlich Habermas (1981, Bd. 1, S. 25–71), Kopperschmidt (1989, S. 15–33).

11 Das von Römer/Stumpf (2018) herausgegebene Themenheft der Zeitschrift *Aptum* gibt einen guten Überblick über die linguistische Erforschung von Verschwörungstheorien. Die Perspektiven anderer Wissenschaften werden in Stumpf/Römer (2020) aufgegriffen.

12 *Narration* sei hier mit Govier/Ayers (2012) verstanden als sinnstiftende Erzählung, in der eine Folge von Ereignissen von einem bestimmten Standpunkt aus geschildert wird. Meist ist dies der Standpunkt einer Person, die diese Ereignisse erlebt hat und die nun als Erzähler fungiert. Typischerweise wird in der Narration ein Problem behandelt, das in irgendeiner Weise einer Lösung zugeführt wird.

13 Vgl. Nocun/Lamberty (2020, S. 21–24), die die Begriffe V*erschwörungsmythos*, -*erzählung* und -*ideologie* bzw. -*mentalität* voneinander abzugrenzen versuchen.

14 Mit Verweis auf historischen wie zeitgenössischen Antisemitismus betonen Nocun/Lamberty (2020, S. 19–21, 106–122) hingegen zu Recht, dass Verschwörungserzählungen durchaus auch Mehrheitsmeinung sein können.

15 Ebenso naheliegend ist eine strukturelle Ähnlichkeit zwischen Verschwörungserzählungen und Kriminalromanen bzw. -filmen, die hier nicht weiterverfolgt werden kann. Sie ergibt sich daraus, dass in Verschwörungserzählungen nicht nur von einer Verschwörung ausgegangen wird, sondern damit gleichzeitig auch immer die Frage nach Verantwortlichen bzw. Schuldigen gestellt und eine Antwort auf die Frage *cui bono?* geliefert wird: »Verschwörungstheoretiker erzählen Geschichte immer vom Ende her. Sie fragen, wem ein Ereignis oder eine Entwicklung nützt, und identifizieren so diejenigen, die dafür verantwortlich sein müssen.« (Butter 2018, S. 59).

16 Eine informative Übersicht zu Verschwörungserzählungen rund um die Corona-Pandemie bieten Nocun/Lamberty (2020, S. 252–274); vgl. zum Folgenden die Zusammenfassung des Redaktionsnetzwerks Deutschland unter https://www.rnd.de/panorama/5-corona-verschworungstheorien-und-ihre-widerlegung-JLA5AH3FB5C7PF7EPJD36RJOD4.html (21.04.2020).

17 https://www.focus.de/politik/deutschland/unterschaetzte-gefahr-rechte-esoteriker-verbreiten-antisemitismus-im-internet_id_11574397.html (22.04.2020). Im September 2020 weist der Youtube-Kanal von Schrang 179.000 Abonnent*innen aus.

18 Die Bilder an der Wand wurden inzwischen ausgetauscht, weil die Witwe John Lennons, Yoko Ono, offenbar gegen die rechtswidrige Nutzung des Fotos geklagt hat; vgl. https://www.g-s-legal.com/yoko-ono-lennon-stoppt-rechtswidrige-foto-nutzung-durch-verschwoerungstheoretiker-heiko-schrang/ (08.11.2020).

19 Alle Videos lassen sich über Schrangs Homepage (https://www.heikoschrang.de/de/startseite/) sowie über seinen Youtube-Kanal (https://www.youtube.com/user/SchrangTV) abrufen. (Bei einer Überprüfung im September 2020 zeigte sich, dass die Videos auf Youtube als »privat« gekennzeichnet wurden, mithin nicht mehr frei zugänglich sind. Auf Schrangs Homepage lassen sich die Videos zwar per Suchfunktion finden, jedoch nicht mehr abspielen.) Für die Analyse wurde in einem ersten Schritt die bei Youtube implementierte Transkriptions-Funktion genutzt, die jedoch insbesondere bei schnell und undeutlich gesprochenen Passagen zu unbefriedigenden Ergebnissen führt. Alle aus den Transkripten zitierten Passagen wurden daher anschließend noch einmal anhand der Videos überprüft und korrigiert. Für Hilfe bei der Korrektur der Transkripte danke ich Frederik Wenning. Textzitate aus den Videos werden anhand des Veröffentlichungsdatums und einer Zeitmarke des Youtube-Videos nachgewiesen und können auf diese Weise überprüft werden.

20 Im Juli 2020 berichtet Spiegel-TV über einen Prozess vor dem Verwaltungsgericht Potsdam, den Schrang, der offenbar seit Jahren keinen Rundfunkbeitrag zahlt, gegen den RBB (Rundfunk Berlin-Brandenburg) anstrengt (https://www.youtube.com/watch?v=zfo-XLu9X0g; 18.09.2020). Zu populistischer Medienkritik von rechts vgl. Niehr (2019b) und Niehr (2020). Medienkritik, die im Rahmen von Corona-Verschwörungstheorien immer wieder zu hören ist, analysieren Römer/Stumpf (2020) am Beispiel eines Videos von Ken Jebsen.

21 Seine Podcasts beginnen stets mit der Formel »Hallo und herzlich willkommen zur neuen Sendung von SchrangTV«. Dabei spricht Schrang seinen eigenen Namen mit deutlich hörbarer Auslautverhärtung (»Schran*k*TV«). Die Podcasts enden mit der Floskel: »Nur wer gegen den Strom schwimmt, der gelangt zur Quelle. Denn nur tote Fische schwimmen mit dem Strom.«.

22 Manchmal finden sich Verweise auf andere »alternative« Medien, denen bescheinigt wird, ebenfalls investigativ zu ermitteln und ihre Rezipient*innen über Verschwörungen aufzuklären.

23 Zu den Schwierigkeiten, Analogie- und Beispielargumentation voneinander abzugrenzen, vgl. Kienpointner (1992, S. 384).

24 Das Zusammenspiel von Analogien, Parabeln und Argumentation beleuchten Govier/Ayers (2012).

25 In diesem Punkt gibt es Berührungspunkte zwischen Verschwörungstheoretikern und esoterischen Kreisen, die sich gerne als »spirituell« bezeichnen. In derartigen »spirituellen« Kreisen gilt die Spruchweisheit »Es gibt keine Zufälle« als nicht hinterfragbares Axiom, das einen verborgenen tieferen Zusammenhang zwischen scheinbar zufälligen Begebenheiten verbürgt, den es zu erkennen gelte. Solche Erkenntnisse seien in erster Linie besonders erleuchteten Menschen vorbehalten, die ihr Wissen sodann an Lernwillige weitergeben.

26 Tatsächlich ist im geltenden Infektionsschutzgesetz (§ 20, Abs. 6) der Bundesrepublik Deutschland bereits jetzt die Möglichkeit zur Einführung einer Impfpflicht gegeben: »Das Bundesministerium für Gesundheit wird ermächtigt, durch Rechtsverordnung mit Zustimmung des Bundesrates anzuordnen, dass bedrohte Teile der Bevölkerung an Schutzimpfungen oder anderen Maßnahmen der spezifischen Prophylaxe teilzunehmen haben, wenn eine übertragbare Krankheit mit klinisch schweren Verlaufsformen auftritt und mit ihrer epidemischen Verbreitung zu rechnen ist. Personen, die auf Grund einer medizinischen Kontraindikation nicht an Schutzimpfungen oder an anderen Maßnahmen der spezifischen Prophylaxe teilnehmen können, können durch Rechtsverordnung nach Satz 1 nicht zu einer Teilnahme an Schutzimpfungen oder an anderen Maßnahmen der spezifischen Prophylaxe verpflichtet werden. § 15 Abs. 2 gilt entsprechend.« (https://www.gesetze-im-internet.de/ifsg/__20.html; 18.09.2020).

27 Zu Herkunft und Bedeutung dieses Schlagwortes vgl. den Wikipedia-Artikel, der bereits im Titel auf verschwörungstheoretische Kontexte aufmerksam macht: https://de.wikipedia.org/wiki/Neue_Weltordnung_(Verschw%C3%B6rungstheorie) (23.11.2020).

28 Die in eckigen Klammern stehende Behauptung stellt die Konklusion der Argumentation dar. Sie wird vor den Prämissen in Klammern aufgeführt, um zu verdeutlichen, dass die Konklusion, nach der es ein ausgemachtes Ziel der Politik sei, das Bargeld abzuschaffen, bei Schrang gar nicht infrage steht und schon vorab als gültig vorausgesetzt wird.

29 Gemeint ist ein rassistischer Anschlag, der im Februar 2020 in der hessischen Stadt Hanau verübt wurde. Zehn Menschen – unter ihnen der Täter und seine Mutter – kamen dabei ums Leben.
